# The Efficacy of Parenteral Nutrition and Enteral Nutrition Supports in Traumatic Brain Injury: A Systemic Review and Network Meta-Analysis

**DOI:** 10.1155/2023/8867614

**Published:** 2023-04-20

**Authors:** Yan Qin, Maoxia Liu, Fengbao Guo, Du Chen, Peng Yang, Xionghui Chen, Feng Xu

**Affiliations:** Department of Emergency Medicine, The First Affiliated Hospital of Soochow University, Suzhou, Jiangsu, China

## Abstract

**Background:**

Enteral nutrition (EN) is often used in patients with traumatic brain injury (TBI), but some studies have shown that EN has its disadvantages. However, it is not clear which nutritional support is appropriate to reduce mortality, improve prognosis, and improve nutritional status in patients with TBI. We performed this Bayesian network meta-analysis to evaluate the improvement of nutritional indicators and the clinical outcomes of patients with TBI.

**Methods:**

We systematically searched PubMed, Embase, Cochrane Library, and Web of Science from inception until December 2021. All randomized controlled trials (RCTs) which compared the effects of different nutritional supports on clinical outcomes and nutritional indicators in patients with TBI were included. The co-primary outcomes included mortality and the value of serum albumin. The secondary outcomes were nitrogen balance, the length of study (LOS) in the ICU, and feeding-related complications. The network meta-analysis was performed to adjust for indirect comparison and mixed treatment analysis.

**Results:**

7 studies enroll a total of 456 patients who received different nutritional supports including parenteral nutrition (PN), enteral nutrition (EN), and PN + EN. No effects on in-hospital mortality (Median RR = 1.06, 95% Crl = 0.12 to 1.77) and the value of 0-1 days of serum albumin were found between the included regimens. However, the value of 11–13 days of serum albumin of EN was better than that of PN (WMD = −4.95, 95% CI = −7.18 to −2.72, *P* < 0.0001, *I*^2^ = 0%), and 16–20 days of serum albumin of EN + PN was better than that of EN (WMD = −7.42, 95% CI = −14.51 to −0.34, *P*=0.04, *I*^2^ = 90%). No effects on the 5–7 day nitrogen balance were found between the included regimens. In addition, the complications including pneumonia and sepsis have no statistical difference between EN and PN. EN was superior to PN in terms of LOS in the ICU and the incidence rate of stress ulcers. Although the difference in indirect comparisons between the included regimens was not statistically significant, the results showed that PN seemed to rank behind other regimens, and the difference between them was extremely small.

**Conclusion:**

Available evidence suggests that EN + PN appears to be the most effective strategy for patients with TBI in improving clinical outcomes and nutritional support compared with other nutritional supports. Further trials are required.

## 1. Introduction

Traumatic brain injury (TBI) is a major global health problem, which is a common presentation in neurosurgery and a leading cause of mortality and disability under the age of 40 [1, 2]. Based on the incidence of traumatic brain injury in China, it is estimated that there are 108 to 332 new hospital admissions per 100,000 people per year [3]. TBI has dynamically changeable life-threatening conditions which impose a heavy burden on socioeconomic consequences [2, 4]. Patients after an injury are always subject to a state of hypermetabolism and hypercatabolism [5]. The increased metabolic rate after TBI has been shown to correlate with intracranial pressure [6]. Increasing the body's basal metabolic rate is a usual way to respond to these changes. The abnormal metabolic processes have been recognized as important elements of secondary injuries. Nutritional support has been appreciated as a crucial treatment for abnormal metabolic conditions following TBI [7]. Moreover, the Brain Trauma Foundation proposed TBI patients should attain basal caloric replacement after injury at least by the fifth day and at most, by the seventh day [8]. But the nutritional support is generally neglected in the TBI population.

TBI patients mainly maintain their nutritional status through enteral nutrition (EN) and parenteral nutrition (PN). Both approaches have their advantages and disadvantages. EN is an important way to correct systemic metabolic disorders, improve immunity, and enhance the poor prognosis of TBI patients, which is in line with human physiological needs. However, intolerance to EN often occurs in the early stage after TBI, and PN is needed to supply the calories required by those patients. However, it should be noted that studies have shown that PN is associated with a higher infection rate, hyperglycemia, hepatic steatosis, and other complications [9].

The Brain Traumatic Foundation has proposed when to attain basal caloric replacement, but there is no agreement about how to reach the optimal route of nutritional support. We perform this network meta-analysis aiming to explore the effect and outcomes of different nutritional supports in TBI patients.

## 2. Methods

### 2.1. Registration

This network meta-analysis is reported in line with the Preferred Reporting Items for Systematic Reviews and Meta-Analyses (PRISMA) statement. The study protocol was registered (identifier: CRD42021292847) on December 24, 2021, with the International Prospective Register of Systematic Reviews (PROSPERO).

### 2.2. Literature Search

Two review authors independently performed the electronic search in PubMed, Embase, Web of Science, and Cochrane Library from inception to December 24, 2021, of which the search strategy was performed around the PICOS. The search strings contain adults with traumatic brain injury, enteral nutrition, parental nutrition, and RCTs. In addition to the databases, the two review authors also independently scanned the reference lists of included studies and included articles that met the inclusion criteria. The complete search strategies are shown in Document S1.

### 2.3. Trial Selection

Trials were included if they met the following eligibility criteria: randomized clinical trials (RCTs) that compared different nutritional supports for patients with TBI; adults (aged 18 years or older) with TBI of any severity or patients with multiple injuries including head injury; any of the treatment strategies of EN, PN, and EN + PN; and the following outcomes: mortality, the value of serum albumin, nitrogen balance, LOS in the ICU, and feeding-associated complications including pneumonia, sepsis, and stress ulcer. Trials were excluded if they met the following exclusion criteria: non-RCT, quasi-randomized trials, and studies based on other languages other than English.

### 2.4. Outcome Measures and Data Extraction

The co-primary outcomes were overall in-hospital mortality and the value of 11–13 days and 16–20 days of serum albumin. The secondary outcomes were nitrogen balance, the LOS in ICU, the incidence rate of pneumonia, the incidence rate of sepsis, and the incidence rate of stress ulcers. Two investigators independently reviewed the included studies and extracted the relevant data from each study, including the year of publication, author's name, region, patients' mean age, patients' sex, Glasgow Coma Scale when admission, sample size, treatment arms, timing to starting nutrition, and the patients' outcomes. Any discrepancies regarding the extraction of data were resolved by recheck of the study data and discussing with the corresponding author. When information is missing, an independent author sought data by sending an email to the original author.

### 2.5. Risk of Bias and Quality Assessment

The methodological quality of individual studies was assessed using the Cochrane risk of the bias assessment tool, based on the following aspects: random sequence generation; allocation concealment; blinding of outcome assessment; incomplete outcome data; selective reporting; and other bias. Each item was assessed with a high, low, or unclear risk of bias, and disagreements were resolved through open discussion with the corresponding authors.

### 2.6. Statistical Analysis

We performed a traditional pairwise meta-analysis using the RevMan software (version 5.4.1, Cochrane Collaboration, Copenhagen, Denmark). For the dichotomous outcomes, relative risks (RRs) with 95% confidence intervals (CIs) were analyzed. For the continuous outcomes, weighted mean difference (WMDs) with 95% CIs was analyzed. Chi-square statistics were used to assess heterogeneity between trials, with an *I*^2^ value greater than 50%, indicating significant heterogeneity [10]. Bayesian network meta-analysis was performed to compare three interventions (EN, PN, and EN + PN) using the automated software GeMTC (version 0.14.3 Groningen, USA). It combines direct and indirect information to obtain estimates of the relative intervention effects of multiple intervention comparisons [11]. The node splitting model was used to evaluate whether direct and indirect effects are consistent. We used noninformative uniform and normal prior distributions and three different sets of starting values to fit the model, yielding 150000 iterations (50000 per chain) to obtain the posterior distributions of model parameters. For overall in-hospital mortality and the value of serum albumin, we used 5000 burn-ins and a thinning interval of 50 for each chain. The rank probability graph generated by the network meta-analysis is designed to find out which nutritional support is best. Random effect variance and inconsistent random effect variance are also used to analyze its consistency.

## 3. Results

### 3.1. Study Selection

**Figure 1:**
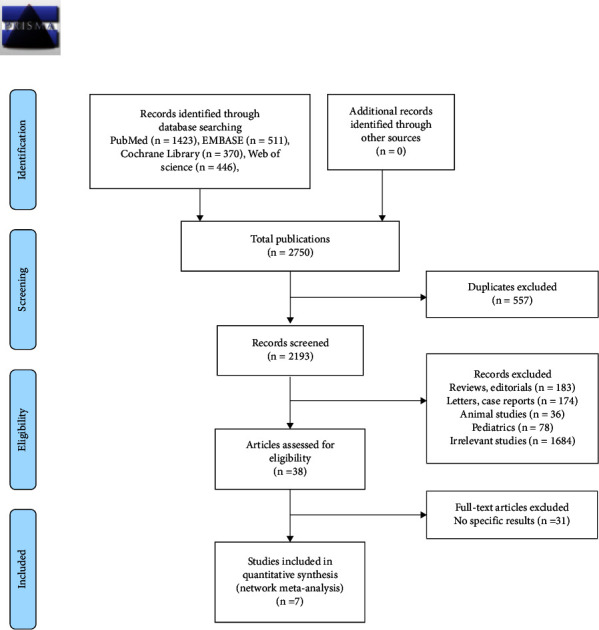
The flowchart of studies evaluating nutritional supports for TBI through the selection process.

The PRISMA diagram is illustrated in Figure 1. We screen PubMed, Embase, Web of Science, and the Cochrane Library from inception to December 24, 2021 and retrieved 2750 articles. We removed 557 duplicate publications, and 2155 articles were excluded according to title and abstract. We performed a further evaluation, and only seven RCTs were included from different countries enrolling 456 patients [12–18].

### 3.2. Study Characteristics

The seven RCTs [12–18] which included 456 individuals with an average age of 37.29 (SD = 8.32) years were conducted in five regions, which included USA (*n* = 2), Iran (*n* = 1), Brazil (*n* = 1), Italy (*n* = 1), and China (*n* = 2), between 1983 and 2016. All the studies included both men and women. And of these, a large proportion is male. The GCS ranges from 3 to 12. The baseline characteristics of the included studies are presented in Table 1. In terms of nutrition pathways, 213 individuals were included in the EN group, 168 individuals in the PN group, and 75 in the EN + PN group.

In relation to outcomes reported, all but three studies [14–16] reported in-hospital mortality, all but three reported the value of serum albumin [13, 15, 16], and all but four reported the nitrogen balance [12, 16–18].

### 3.3. Risk of Bias

**Figure 2:**
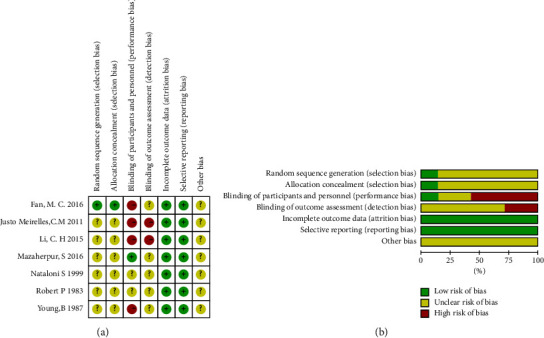
Quality assessment of included studies.

Details of the quality assessment in each study included are provided in Figure 2. A judgment of low, high, or unclear risk of bias was rated in each domain, and then the study was evaluated to be at a low risk of bias (if all domains were at low risk), a high risk of bias (if high risk in ≥ 1 domain), or unclear risk of bias (if unclear risk in ≥ 1 domain without any domain at a high risk). Of these, 0 RCTs had a low risk of bias, 3 had an unclear risk of bias, and 4 had a high risk of bias.

## 4. Results from Direct Comparisons

### 4.1. Primary Outcomes

**Figure 3:**
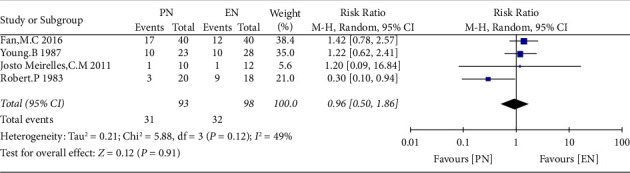
The direct comparison of in-hospital mortality between the EN group and the PN group.

The primary outcomes of direct comparisons are the in-hospital mortality and the value of serum albumin during 11–13 days after EN, PN, and EN + PN support. In-hospital mortality had no significant difference in the EN group when compared with the PN group (RR = 0.96, 95% CI = 0.50 to 1.86, *P*=0.91, and *I*^2^ = 49%). (Figure 3)

**Figure 4:**
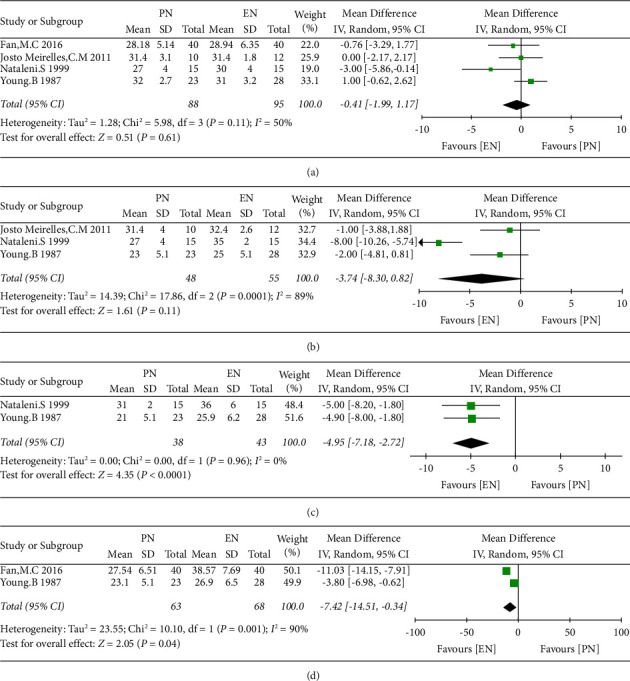
The value of serum albumin during 0-1 d (a), 5–7 d (b), 11–13 d (c), and 16–20 d (d) between the EN and PN groups.

There was obvious heterogeneity among these included studies; thus, the random effect model was utilized for statistical analysis. The results indicated that the value of serum albumin had no significant difference during 0-1 days and 5–7 days but increased during 11–13 days (WMD = −4.95, 95% CI = −7.18 to −2.72, *P* < 0.0001, and *I*^2^ = 0%) and 16–20 days (WMD = −7.42, 95% CI = −14.51 to −0.34, *P*=0.04, and *I*^2^ = 90%) in the EN group compared to the PN group (Figure 4)

### 4.2. Secondary Outcomes

Between the EN group and the PN group, there was an increase trend of the LOS in the ICU in the PN group (WMD = 3.98, 95% CI = 0.31 to 7.65, *P*=0.03, and *I*^2^ = 13%) (Figure S1). Moreover, three complications were analyzed, including pneumonia, sepsis, and stress ulcer. Incidence of stress ulcer in the PN group was higher than the EN group (RR = 2.97, 95% CI = 1.47 to 6.00, *P*=0.002, and *I*^2^ = 0%) (Figure S2). Compared with the PN group, the EN group did not significantly reduce the incidence of pneumonia (RR = 0.83, 95% CI = 0.38 to 1.78, *P*=0.63, and *I*^2^ = 52%) and sepsis (RR = 2.36, 95% CI = 0.28 to 20.12, *P*=0.43, and *I*^2^ = 62%). And in contrast to the EN group, the EN + PN group had no influence on the value of serum albumin during 0 days (*P*=0.61).

We compared nitrogen balance between the PN and the EN groups and between the EN + PN and the EN groups, respectively. There was no heterogeneity in nitrogen balance during 10-11 days between EN and PN groups, and the nitrogen balance during 10-11 days in the EN group was higher than in the PN group (WMD = −3.64; 95% CI = −4.77 to −2.50, *P* < 0.00001, and *I*^2^ = 0%) (Figure S3). Random effect models were utilized (*I*^2^ > 75%) in the five outcomes (nitrogen balance during 0-1 days, 3 days, 7 days in PN vs. EN group (Figure S3); nitrogen balance during 7 days, 11–14 days in EN + PN vs. EN group). The results showed that there was no statistically significant difference on all of these (Figure S4).

### 4.3. Results from the Network Meta-analysis (NMA)

**Figure 5:**
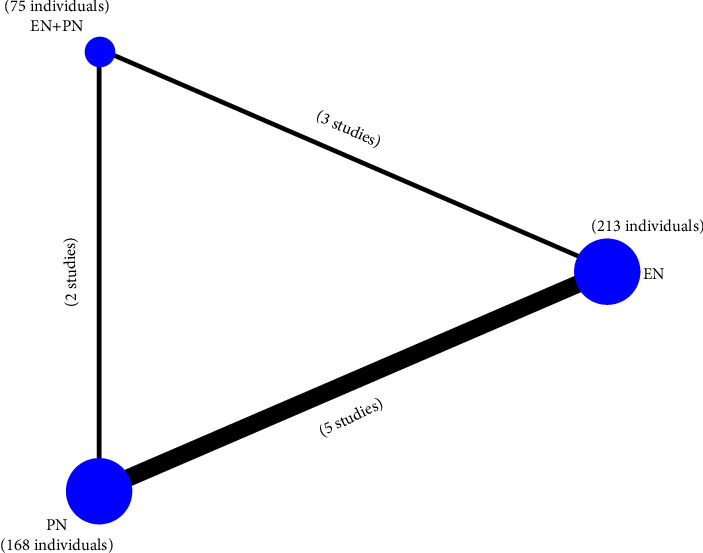
The comparison network of the included studies.

**Figure 6:**
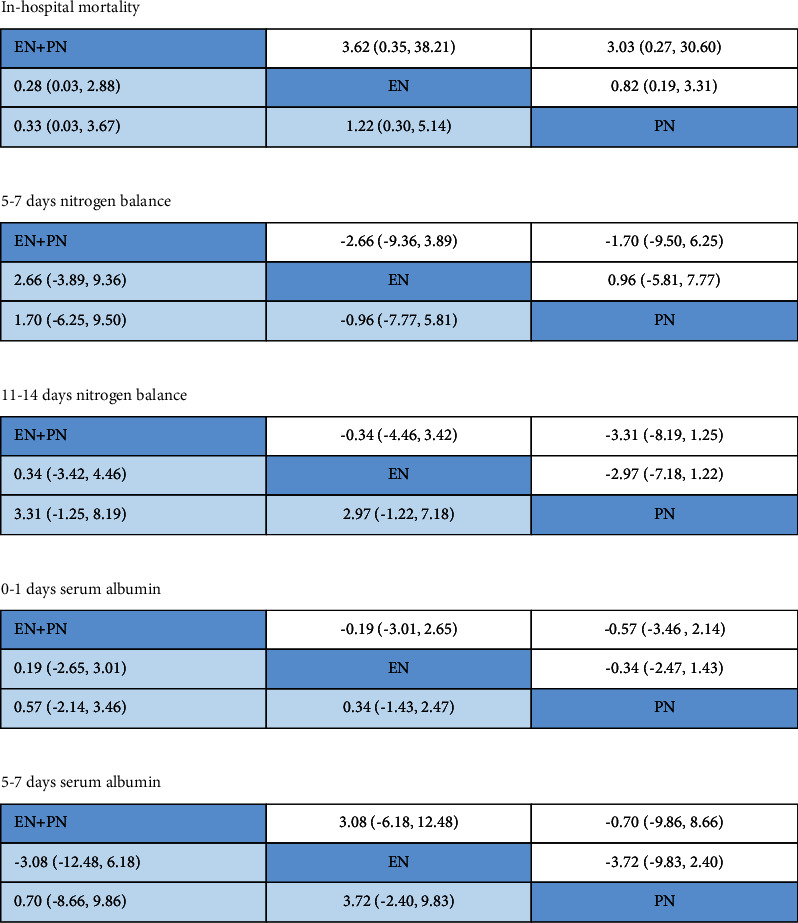
The matrix for the results of the in-hospital mortality, 5–7 days nitrogen balance, 11–14 days nitrogen balance, 0-1 days serum albumin, and 5–7 days serum albumin.

All networks held the principles of coherency, transitivity, and consistency. NMA maps of the studies examining the in-hospital mortality and the value of serum albumin of different nutritional supports are in Figure 5. The size of the nodes relates to the number of individuals in that intervention type. 213 individuals were included in the EN group, 168 individuals in the PN group, and 75 in the EN + PN group. The thickness of lines between interventions relates to the number of studies for that comparison. There are 3 studies relates to EN + PN vs. EN, 5 studies related to EN vs. PN, and 2 studies related to EN + PN vs. PN.  Figure 6 details the complete matrix for results of the in-hospital mortality, 5–7 days nitrogen balance, 11–14 days nitrogen balance, 0-1 days serum albumin, and 5–7 days serum albumin. And Table 2 ranks the nutritional pathways based on the outcome being measured.

Four studies with 231 individuals contributed to the NMA assessing the in-hospital mortality. There is no significant difference in the in-hospital mortality in the three groups. But Table 2 illustrates that the EN + PN group may rank the best for achieving a higher survival rate.  Figure 6 illustrates the complete matrix.

Four studies with 238 individuals contributed to the analysis of the value of serum albumin. There is no significant difference in the value of serum albumin on 0-1 days or 5–7 days after admission in the three groups. The rank probability of albumin showed that the probability of the EN + PN group ranking the first was higher than that in the PN group and the EN group on 0-1 days after admission, while the probability of the EN + PN group ranking the first was higher than that in the EN group and the PN group on 5–7 days after admission. The figure illustrates the complete matrix.

## 5. Discussion

The systemic metabolism rate of a patient with TBI is usually disrupted, and the energy consumption is usually faster than those of a normal person. These changes lead to the process of undernutrition complications such as negative nitrogen balance and hypoproteinemia, which escalate brain damage and poor prognosis. Although it is known that nutritional support cannot completely reverse the catabolic state [19], appropriate nutritional support can effectively enhance the nutritional status and have a great significant effect on the recovery of patients.

This NMA represents the analysis regarding nutritional interventions for people with TBI. We combined direct and indirect evidence from 7 RCTs comparing 3 different intervention arms in over 456 adults with TBI. Our main outcomes indicate that a combined intervention consisting of the PN and the EN may be promising nutrition support for decreasing in-hospital mortality in patients with TBI. However, this conclusion is not statistically significant, which may be due to the small sample size, early studies, and the few relevant RCTs, which might contribute to the discrepancy. From other studies [20, 21], these findings are supported, as while there is some cross-over between the benefits of PN and EN; each also contributes especially to the body's recovery after injury. Studies note that gastrointestinal function impairment is common in patients with TBI [22]. PN can rapidly improve the nutritional status of patients in a short period and is superior to EN in early life-saving nutritional support. But at the same time, the timely opening of the EN is more in line with the physiological needs, is the more effective use of substrates to better support cell and organ functions, can avoid liver immune injury induced by PN, and can reduce the occurrence of complications such as hyperglycemia and hyperosmia. Therefore, it is reasonable to assume that these two nutritional supports together contribute to further improving the outcome of TBI patients, thereby reducing patient in-hospital mortality.

After the TBI, the value of serum albumin may decrease due to increased consumption, bleeding loss, insufficient intake, or other reasons. Hypoproteinemia is bound to weaken the patient's resistance and pose a risk of poor prognosis [23]. It is now generally accepted that a moderately high level of serum albumin may be most beneficial for patients with TBI [3]. Appropriate nutritional support to improve serum albumin is particularly important. Our direct results showed that EN and EN + PN groups had no significant difference in the value of serum albumin at day 0, while the value of serum albumin in the EN + PN group on days 16–20 was significantly higher than the EN group, indicating that EN + PN had a better effect on improving serum albumin than EN. NMA results showed that no matter at 0-1 day or 5–7 days, three groups of the value of serum albumin had no obvious difference. The emergence of this outcome may be due to the following reasons: albumin has a longer half-life (about 20 days), and it is a negative acute-phase reactant, meaning that serum albumin concentrations rise slowly during nutritional therapy [3, 24]. Therefore, it is necessary to extend the study period when assessing nutritional status improvement with serum albumin. A study by Caliri et al. [25] showed that the initial serum albumin value of patients with TBI was much lower than the minimum normal level on admission. And using enteral caloric implementation, hypoalbuminemia improved slightly after 1 month, and serum albumin returned to normal after 12 months. In our analysis, the included articles have insufficient data to support more days of serum albumin in this analysis. However, according to the rank probability results of NMA, EN may rank the first in improving the serum albumin through 5–7 days of nutritional support. Therefore, combined with the results of direct comparison and network analysis, the EN (with or without the PN) is more valuable than the PN alone for increasing serum albumin, a reliable nutritional indicator. But, it is worth noting that the use of fluid resuscitation with albumin for TBI in the first week may increase intracranial pressure, which is the most likely mechanism of increased mortality in these patients [26]. We should also note that albumin is not recommended for resuscitation in patients with TBI [27].

Improved nitrogen balance is associated with increased protein intake. Nitrogen loss is not only related to nitrogen input but also the catabolic rate. Clinicians often use this indicator to assess the catabolic status and adjust nutritional regimens in response to nitrogen balance changes. We notice that other studies proposed that nitrogen balance was independently associated with improved survival [28]. In this study, nitrogen balance was analyzed to evaluate the impact of different nutritional supports on the nutritional status of TBI patients. Our direct results indicated that there was no significant difference in nitrogen balance at 0-1 day between the EN group and the PN group, but the nitrogen balance at 10-11 days of the EN group was higher than that of the PN group, indicating that EN had a better effect on improving nitrogen balance than PN. Our NMA analysis results showed that there was no statistically significant difference in nitrogen balance between the EN group, PN group, and EN + PN group at 5–7 days nor between 11-14 days. However, it was worth noting that the ranking of nitrogen balance at 5–7 days and 11–14 days showed that with the extension of nutritional intervention time, up to 11–14 days, the order of nitrogen balance from the initial PN > EN changed to EN > PN, but the effect of EN + PN and EN on the improvement of nitrogen balance cannot be judged. In ranking nutritional interventions to decrease mortality and improve nitrogen balance, the 5–7 day PN group was deemed to be the least effective. We notice that some studies prove that PN can improve nitrogen balance rapidly. It helps to improve lymphocyte levels quickly, which is conducive to the recovery of immune function [29]. So, the PN also cannot be ignored in the treatment of TBI.

Our meta-analysis results showed that the LOS in the ICU and the incidence of stress ulcer in the EN group were significantly lower than those in the PN group, and there was no statistical difference in the pneumonia rate. Making a scientific and reasonable plan to ensure the smooth implementation of early enteral nutrition can not only maintain the barrier function of the gastrointestinal tract but also prevent intestinal toxins from entering the bloodstream and causing bacterial translocation [30–33]. In addition, earlier studies believed that the EN was closely related to the occurrence of aspiration pneumonia, but in recent years, due to the popularity of nasojejunal feeding, percutaneous endoscopic gastrostomy feeding, and transpyloric feeding, the incidence of aspiration pneumonia has decreased significantly [34–36]. In addition, it is undeniable that the support of the PN in the period of intolerance to the EN can improve the immune function of patients with TBI and promote recovery. In neurosurgery, patients with TBI have a high rate of mortality and disability rate, and its treatment is extremely challenging. Every link of treatment, including nutrition, should not be ignored.

In this study, only English-language articles were searched, which may lead to incomplete article collection and may reduce the quality of research results. In addition, we realize that positive results may be easier to publish than negative results, which resulted in inherent publication bias. Moreover, the sample sizes are relatively small, and the heterogeneities in sample and methodology are inherent across the included articles, so nutritional supports cannot be studied in detail. Too many risks of bias are unclear when we assessed the risk of bias, which may skew the results of the network meta-analysis from the truth. We recommend that more high-quality RCTs be conducted in the future to guide clinical work. All of these limitations may affect the authenticity of conclusions.

## 6. Conclusions

Available evidence suggests that EN + PN appears to be the most effective strategy for patients with TBI in improving clinical outcomes and nutritional support compared with other nutritional supports. Further trials are required.

## Figures and Tables

**Table 1:** Characteristics of included studies.

Author (year)	Region	Sample size	Treatment arms (no.)	Mean age (*y*)	Sex (male), (%)	GCS (admission)	Timing to starting of nutrition	Outcomes
Young, 1987	USA	51	Early PN (23) vs. delayed EN (28)	33.0	82.4	4–10	Early PN: within 48 hours postinjury; delayed EN: till the termination of low wall suction	GOS; mortality; complication rate; nitrogen balance; caloric balance; nitrogen intake; lymphocyte counts; albumin levels
Mazaherpur, 2016	Iran	60	Continuous EN (20) vs. intermittent EN (20) vs. combination EN + PN (20)	34.6	76.7	3–8	NA	Received energy and protein; nitrogen balance; anthropometric indicators (weight; IBM)
Robert, 1983	USA	38	Early TPN (20) vs. delayed SEN (18)	40	NA	Mean: 7.5	Early PN: within 48 h after admission; delayed EN: bowel sounds were present and the gastric residual volume was less than 100 ml/hour	Mortality; GOS; nitrogen balance; nitrogen intake; calorie intake; serum transferrin values; serum albumin; serum glucose level; body temperature; triceps skinfold; midarm muscle circumference; total lymphocyte count; any other complication
Justo Meirelles, 2011	Brazil	22	EN (12) vs. TPN (10)	31	90.9	9–12	Hemodinamically stable	Mortality; morbidity; length of stay in ICU; days of mechanical ventilation; calories and protein received daily; glucose, albumin, urea, creatinine, C-reactive protein (CRP), the ratio CRP/albumin; urinary urea nitrogen; nitrogen balance; N ingested-N excreted
Nataloni, 1999	Italy	45	EN (15) vs. PN (15) vs. EN + PN (15)	28	68.9	Mean: 5.5	All patients: two days after ICU admission	Serum prealbumin, RBP, albumin, transferrin, nitrogen balance
Li, 2015	CN	120	Early EN (60) vs. PN (60)	52.7	53.3	NA	All patients: starting 48 h after surgery	Complication rate, albumin (ALB), alanine aminotransferase (ALT), blood glucose (GLU) level, monitoring time and cost
Fan, 2016	CN	120	EN (40) vs. PN (40) vs. early EN + PN (40)	41.7	51.3	6–8	All patients: within 48 h after admission	Mortality, T lympholeukocyte subsets, plasma immunoglobulin, serum total protein, serum albumin, serum prealbumin and hemoglobin, complications, length of stay in the NICU, mechanical ventilator utilization rate, and its durations

**Table 2:** Results of the rank test for different nutritional supports.

Supports	Rank 1	Rank 2	Rank 3
*In-hospital mortality*			
EN + PN	0.08	0.1	0.82
EN	0.58	0.35	0.07
PN	0.34	0.54	0.12

*0-1 days serum albumin*			
EN + PN	0.51	0.24	0.26
EN	0.33	0.41	0.26
PN	0.17	0.35	0.48

*5–7 days serum albumin*			
EN + PN	0.2	0.38	0.41
EN	0.74	0.21	0.05
PN	0.06	0.4	0.54

*5–7 days nitrogen balance*			
EN + PN	0.62	0.25	0.13
EN	0.11	0.36	0.54
PN	0.27	0.39	0.34

*11–14 days nitrogen balance*			
EN + PN	0.56	0.38	0.06
EN	0.41	0.53	0.06
PN	0.03	0.09	0.88

## Data Availability

The data supporting the findings of the study can be obtained from the corresponding author upon request.

## Supplementary Materials

Supplementary Materials Figure S1: LOS in the ICU between the EN and PN groups. Figure S2: Incidence of stress ulcer between the EN and PN groups. Figure S3: Nitrogen balance during 0-1 d (A), 3 d (B), 7 d (C), and 10-11 d (D) between the EN and PN groups. Figure S4: Nitrogen balance during 7 d (A) and 11–14 d (B) between the EN + PN and EN groups. Document S1: Details of the search strategy.

## References

[1] Czeiter E., Mondello S., Kovacs N. (2012). Brain injury biomarkers may improve the predictive power of the IMPACT outcome calculator. *Journal of Neurotrauma*.

[2] Rakhit S., Nordness M. F., Lombardo S. R., Cook M., Smith L., Patel M. B. (2021). Management and challenges of severe traumatic brain injury. *Seminars in Respiratory and Critical Care Medicine*.

[3] Chen D., Bao L., Lu S. Q., Xu F. (2014). Serum albumin and prealbumin predict the poor outcome of traumatic brain injury. *PLoS One*.

[4] Wijayatilake D. S., Shepherd S. J., Sherren P. B. (2012). Updates in the management of intracranial pressure in traumatic brain injury. *Current Opinion in Anaesthesiology*.

[5] Wilson R. F., Tyburski J. G. (1998). Metabolic responses and nutritional therapy in patients with severe head injuries. *The Journal of Head Trauma Rehabilitation*.

[6] Bucci M. N., Dechert R. E., Arnoldi D. K., Campbell J., McGillicuddy J. E., Bartlett R. H. (1988). Elevated intracranial pressure associated with hypermetabolism in isolated head trauma. *Acta Neurochirurgica*.

[7] Borzotta A. P., Pennings J., Papasadero B. (1994). Enteral versus parenteral nutrition after severe closed head injury. *The Journal of Trauma, Injury, Infection, and Critical Care*.

[8] Carney N., Totten A. M., O’Reilly C. (2017). Guidelines for the management of severe traumatic brain injury, fourth edition. *Neurosurgery*.

[9] Jeejeebhoy K. N. (2001). Enteral and parenteral nutrition: evidence-based approach. *Proceedings of the Nutrition Society*.

[10] Higgins J. P., Chandler J., Cumpston M., Li T., Page M. J., Welch V. A. (2008). *Cochrane Handbook For Systematic Reviews of Interventions*.

[11] Chaimani A. C. D., Li T., Higgins J. P. T., Salanti G. (2022). Cochrane handbook for systematic reviews of interventions version 6.3. *Chapter 11: Undertaking network meta-analyses*.

[12] Young B., Ott L., Twyman D. (1987). The effect of nutritional support on outcome from severe head injury. *Journal of Neurosurgery*.

[13] Rapp R. P., Pharm D., Young B. (1983). The favorable effect of early parenteral feeding on survival in head-injured patients. *Journal of Neurosurgery*.

[14] Nataloni S., Gentili P., Marini B. (1999). Nutritional assessment in head injured patients through the study of rapid turnover visceral proteins. *Clinical Nutrition*.

[15] Mazaherpur S., Khatony A., Abdi A., Pasdar Y., Najafi F. (2016). The effect of continuous enteral nutrition on nutrition indices, compared to the intermittent and combination enteral nutrition in traumatic brain injury patients. *Journal of Clinical and Diagnostic Research*.

[16] Li C. H., Chen D. P., Yang J. (2015). Enteral nutritional support in patients with head injuries after craniocerebral surgery. *Turk Neurosurg*.

[17] Justo Meirelles C. M., de Aguilar-Nascimento J. E. (2011). Enteral or parenteral nutrition in traumatic brain injury: a prospective randomised trial. *Nutricion Hospitalaria*.

[18] Fan M. C., Wang Q. L., Fang W. (2016). Early enteral combined with parenteral nutrition treatment for severe traumatic brain injury: effects on immune function, nutritional status and outcomes. *Chinese Medical Sciences Journal*.

[19] Frankenfield D. C., Smith J. S., Cooney R. N. (1997). Accelerated nitrogen loss after traumatic injury is not attenuated by achievement of energy balance. *JPEN - Journal of Parenteral and Enteral Nutrition*.

[20] Cook A. M., Peppard A., Magnuson B. (2008). Nutrition considerations in traumatic brain injury. *Nutrition in Clinical Practice*.

[21] Wang X., Dong Y., Han X., Qi X.-Q., Huang C.-G., Hou L.-J. (2013). Nutritional support for patients sustaining traumatic brain injury: a systematic review and meta-analysis of prospective studies. *PLoS One*.

[22] Hanscom M., Loane D. J., Shea-Donohue T. (2021). Brain-gut axis dysfunction in the pathogenesis of traumatic brain injury. *The Journal of Clinical Investigation*.

[23] Bernard F., Al-Tamimi Y. Z., Chatfield D., Lynch A. G., Matta B. F., Menon D. K. (2008). Serum albumin level as a predictor of outcome in traumatic brain injury: potential for treatment. *The Journal of Trauma, Injury, Infection, and Critical Care*.

[24] Collins N. (2001). The difference between albumin and prealbumin. *Advances in Skin and Wound Care*.

[25] Caliri S., Andaloro A., Corallo F. (2019). Recovery of malnutrition in a patient with severe brain injury outcomes: a case report. *Medicine (Baltimore)*.

[26] Cooper D. J., Myburgh J., Heritier S. (2013). Albumin resuscitation for traumatic brain injury: is intracranial hypertension the cause of increased mortality?. *Journal of Neurotrauma*.

[27] Abdelmalik P. A., Draghic N., Ling G. S. F. (2019). Management of moderate and severe traumatic brain injury. *Transfusion*.

[28] Frankenfield D. (2006). Energy expenditure and protein requirements after traumatic injury. *Nutrition in Clinical Practice*.

[29] Palesty J. A., Dudrick S. J. (2011). Cachexia, malnutrition, the refeeding syndrome, and lessons from Goldilocks. *Surgical Clinics of North America*.

[30] Reintam Blaser A., Starkopf J., Alhazzani W. (2017). Early enteral nutrition in critically ill patients: ESICM clinical practice guidelines. *Intensive Care Medicine*.

[31] Tan M., Zhu J. C., Yin H. H. (2011). Enteral nutrition in patients with severe traumatic brain injury: reasons for intolerance and medical management. *British Journal of Neurosurgery*.

[32] Chourdakis M., Kraus M. M., Tzellos T. (2012). Effect of early compared with delayed enteral nutrition on endocrine function in patients with traumatic brain injury: an open-labeled randomized trial. *JPEN - Journal of Parenteral and Enteral Nutrition*.

[33] Li X., Yang Y., Ma Z. F. (2022). Enteral combined with parenteral nutrition improves clinical outcomes in patients with traumatic brain injury. *Nutritional Neuroscience*.

[34] Grahm T. W., Zadrozny D. B., Harrington T. (1989). The benefits of early jejunal hyperalimentation in the head-injured patient. *Neurosurgery*.

[35] Kostadima E., Kaditis A. G., Alexopoulos E. I., Zakynthinos E., Sfyras D. (2005). Early gastrostomy reduces the rate of ventilator-associated pneumonia in stroke or head injury patients. *European Respiratory Journal*.

[36] Acosta-Escribano J., Fernández-Vivas M., Grau Carmona T. (2010). Gastric versus transpyloric feeding in severe traumatic brain injury: a prospective, randomized trial. *Intensive Care Medicine*.

