# Supplementary material for: The Efficacy of Parenteral Nutrition and Enteral Nutrition Supports in Traumatic Brain Injury: A Systemic Review and Network Meta-Analysis
Source: Emerg Med Int. 2023 Apr 20;2023:8867614. doi: 10.1155/2023/8867614 (PMC10139805; doi:10.1155/2023/8867614)
Supplement: Supplementary Materials — Figure S1: LOS in the ICU between the EN and PN groups. Figure S2: Incidence of stress ulcer between the EN and PN groups. Figure S3: Nitrogen balance during 0-1 d (A), 3 d (B), 7 d (C), and 10-11 d (D) between the EN and PN groups. Figure S4: Nitrogen balance during 7 d (A) and 11–14 d (B) between the EN + PN and EN groups. Document S1: Details of the search strategy. [file 8867614.f1.zip › Document S1.docx]

**Details of the search strategy**

**the Cochrane Central Register of Controlled Trials (CENTRAL)**

#1 MeSH descriptor: [Enteral Nutrition] explode all trees

#2 (enteric feeding):ti,ab,kw OR (nutritional support):ti,ab,kw OR (nutritio* support):ti,ab,kw OR (enteral feed*):ti,ab,kw OR (tube feed*):ti,ab,kw (Word variations have been searched)

#3 (nasogastric):ti,ab,kw OR (nasoenteral):ti,ab,kw OR (oral feed*):ti,ab,kw OR (sip feed*):ti,ab,kw OR (gastrostomy tube*):ti,ab,kw (Word variations have been searched)

#4 (jejunostomy tube*):ti,ab,kw OR (gastric feed*):ti,ab,kw OR (Nutrition Enteral):ti,ab,kw OR (feeding tube*):ti,ab,kw OR (enteral formula):ti,ab,kw (Word variations have been searched)

#5 (EN):ti,ab,kw (Word variations have been searched)

#6 #1 OR #2 OR #3 OR #4 OR #5

#7 MeSH descriptor: [Parenteral Nutrition] explode all trees

#8 (nutrition, parenteral):ti,ab,kw OR (feed*, parenteral):ti,ab,kw OR (parenteral feed*):ti,ab,kw OR (feed*, intravenous):ti,ab,kw OR (intravenous feed*):ti,ab,kw (Word variations have been searched)

#9 (PN):ti,ab,kw OR (TPN):ti,ab,kw (Word variations have been searched)

#10 (Parenteral Nutrition, Total):ti,ab,kw OR (Parenteral Hyperalimentation):ti,ab,kw OR (Total Parenteral Nutrition):ti,ab,kw OR (Hyperalimentation, Parenteral):ti,ab,kw OR (Nutrition, Total Parenteral):ti,ab,kw (Word variations have been searched)

#11 (Intravenous Hyperalimentation):ti,ab,kw OR (Hyperalimentation, Intravenous):ti,ab,kw (Word variations have been searched)

#12 #7 OR #8 OR #9 OR #10 OR #11

#13 (NUTRITION):ti,ab,kw OR (NUTRITION THERAPY):ti,ab,kw (Word variations have been searched)

#14 #12 OR #13 OR #6

#15 MeSH descriptor: [Brain Injuries, Traumatic] explode all trees

#16 (tbi* (traumatic brain injur*)):ti,ab,kw OR (traumatic encephalopathy):ti,ab,kw OR (injury, brain, traumatic):ti,ab,kw OR (traumatic encephalopath*):ti,ab,kw OR (encephalopath*, traumatic):ti,ab,kw (Word variations have been searched)

#17 (trauma*, brain):ti,ab,kw OR (brain trauma*):ti,ab,kw OR (traumatic brain injur*):ti,ab,kw OR (traumatic brain injury):ti,ab,kw OR (brain injur*, traumatic):ti,ab,kw (Word variations have been searched)

#18 MeSH descriptor: [Brain Injuries] explode all trees

#19 MeSH descriptor: [Craniocerebral Trauma] explode all trees

#20 (injur*, Brain):ti,ab,kw OR (Brain Laceration*):ti,ab,kw OR (Laceration*, Brain):ti,ab,kw OR (Trauma, Cerebrovascular):ti,ab,kw OR (Brain Vascular Trauma):ti,ab,kw (Word variations have been searched)

#21 (head injur*):ti,ab,kw OR (Cerebrovascular Trauma):ti,ab,kw OR (Trauma, Cerebrovascular):ti,ab,kw OR (diffuse axonal injur*):ti,ab,kw (Word variations have been searched)

#22 #15 OR #16 OR #17 OR #18 OR #19 OR #20 OR #21

#23 #22 AND #14

#24 (child*):ti,ab,kw OR (pediatric*):ti,ab,kw OR (infant*):ti,ab,kw OR (infant*, newborn):ti,ab,kw OR (newborn infant*):ti,ab,kw (Word variations have been searched)

#25 (neonat*):ti,ab,kw (Word variations have been searched)

#26 #24 or #25

#27 (animal*):ti,ab,kw OR (mice):ti,ab,kw OR (rat):ti,ab,kw OR (rabbit*):ti,ab,kw (Word variations have been searched)

#28 #23 NOT #26

#29 #28 NOT #27

**PubMed search strategy**

((((((((((((((((((((((enteric feeding) OR (nutritional support)) OR (enteral nutritio*)) OR (enteral feed*)) OR (tube feed*)) OR (nutritio* support)) OR (nasogastric)) OR (nasoenteral)) OR (oral feed*)) OR (sip feed*)) OR (gastrostomy tube*)) OR (jejunostomy tube*)) OR (gastric feed*)) OR (Nutrition Enteral)) OR (feeding tube*)) OR (enteral formula)) OR (EN)) OR (((((((((parenteral nutrition) OR (nutrition, parenteral)) OR (feed*, parenteral)) OR (parenteral feed*)) OR (feed*, intravenous)) OR (intravenous feed*)) OR (PN)) OR (TPN)) OR (((((((Parenteral Nutrition, Total) OR (Parenteral Hyperalimentation)) OR (Total Parenteral Nutrition)) OR (Hyperalimentation, Parenteral)) OR (Nutrition, Total Parenteral)) OR (Intravenous Hyperalimentation)) OR (Hyperalimentation, Intravenous)))) OR ((Nutrition) OR (Nutrition Therapy))) AND (((((((((((((tbi* (traumatic brain injur*)) OR (traumatic encephalopathy)) OR (injury, brain, traumatic)) OR (traumatic encephalopath*)) OR (encephalopath*, traumatic)) OR (trauma*, brain)) OR (brain trauma*)) OR (traumatic brain injur*)) OR (brain injur*, traumatic)) OR (traumatic brain injury)) OR ((((brain injur*) OR (Injur*, Brain)) OR (Brain Laceration*)) OR (Laceration*, Brain))) OR (((Cerebrovascular trauma) OR (Trauma, Cerebrovascular)) OR (Brain Vascular Trauma))) OR ((((head injur*) OR (Craniocerebral Trauma)) OR (Cerebrovascular Trauma)) OR (diffuse axonal injur*)))) AND ((clinical[tiab] AND trial[tiab]) OR "clinical trials as topic"[mesh] OR "clinical trial"[pt] OR random*[tiab] OR "random allocation"[mesh] OR "therapeutic use"[sh])) NOT (((((((child) OR (children)) OR (pediatric*)) OR (infant*)) OR (infant*, newborn)) OR (newborn infant*)) OR (neonat*))) NOT ((((animal*) OR (mice)) OR (rat)) OR (rabbit*))

**Embase search strategy**

#64. #40 AND #62 AND #63

#63. 'clinical':ti,ab AND 'trial':ti,ab OR 'clinical trial'/exp OR random* OR 'drug therapy':lnk

#62. #41 OR #42 OR #43 OR #44 OR #45 OR #46 OR #47 OR

#48 OR #49 OR #50 OR #51 OR #52 OR #53 OR #54 OR

#55 OR #56 OR #57 OR #58 OR #59 OR #60 OR #61

#61. 'diffuse axonal injur*':ti,ab

#60. 'cerebrovascular trauma':ti,ab

#59. 'craniocerebral trauma':ti,ab

#58. 'head injur*':ti,ab

#57. 'brain vascular trauma':ti,ab

#56. 'trauma, cerebrovascular':ti,ab

#55. 'cerebrovascular trauma'/exp

#54. 'laceration*, brain':ti,ab

#53. 'brain laceration*':ti,ab

#52. 'injur*, brain':ti,ab

#51. 'brain injur*':ti,ab

#50. 'tbi* (traumatic brain injur*)':ti,ab

#49. 'traumatic encephalopathy':ti,ab

#48. 'injury, brain, traumatic':ti,ab

#47. 'traumatic encephalopath*':ti,ab

#46. 'encephalopath*, traumatic':ti,ab

#45. 'trauma*, brain':ti,ab

#44. 'brain trauma*':ti,ab

#43. 'traumatic brain injur*':ti,ab

#42. 'brain injur*, traumatic':ti,ab

#41. 'traumatic brain injury'/exp

#40. #18 OR #38 OR #39

#39. #27 OR #35

#38. #36 OR #37

#37. 'nutrition therapy':ab,ti

#36. 'nutrition':ab,ti

#35. #28 OR #29 OR #30 OR #31 OR #32 OR #33 OR #34

#34. 'hyperalimentation, intravenous':ab,ti

#33. 'intravenous hyperalimentation':ab,ti

#32. 'nutrition, total parenteral':ab,ti

#31. 'hyperalimentation, parenteral':ab,ti

#30. 'total parenteral nutrition':ab,ti

#29. 'parenteral hyperalimentation':ab,ti

#28. 'parenteral nutrition, total'/exp

#27. #19 OR #20 OR #21 OR #22 OR #23 OR #24 OR #25 OR

#26. 'tpn':ab,ti

#25. 'pn':ab,ti

#24. 'intravenous feed*':ab,ti

#23. 'feed*, intravenous':ab,ti

#22. 'parenteral feed*':ab,ti

#21. 'feed*, parenteral':ab,ti

#20. 'nutrition, parenteral':ab,ti

#19. 'parenteral nutrition'/exp

#18. #1 OR #2 OR #3 OR #4 OR #5 OR #6 OR #7 OR #8 OR

#9 OR #10 OR #11 OR #12 OR #13 OR #14 OR #15 OR

#16 OR #17

#17. 'en':ab,ti

#16. 'enteral formula':ab,ti

#15. 'feeding tube*':ab,ti

#14. 'nutrition enteral':ab,ti

#13. 'gastric feed*':ab,ti

#12. 'jejunostomy tube*':ab,ti

#11. 'gastrostomy tube*':ab,ti

#10. 'sip feed*':ab,ti

#9. 'oral feed*':ab,ti

#8. 'nasoenteral':ab,ti

#7. 'nasogastric':ab,ti

#6. 'nutritio* support':ab,ti

#5. 'tube feed*':ab,ti

#4. 'enteral feed*':ab,ti

#3. 'enteral nutritio*':ab,ti

#2. 'nutritional support'/exp

#1. 'enteric feeding'/exp

**Web of Science search strategy**

1# TS=(enteric feeding OR nutritional support OR enteral nutrition* OR enteral feed* OR tube feed* OR nutritional support OR nutrition support OR Support, Nutritional OR nasogastric OR nasoenteral OR oral feed* OR sip feed* OR tube feed* OR gastrostomy tube* OR jejunostomy tube* OR jejunostomy tube* OR Nutrition Enteral OR feeding tube* OR enteral formula OR EN)

2# TS=(parenteral nutrition OR nutrition, parenteral OR feeding*, parenteral OR parenteral feeding* OR feeding*, intravenous OR intravenous feeding* OR PN OR TPN OR Parenteral Nutrition, Total OR Parenteral Hyperalimentation OR Total Parenteral Nutrition OR Hyperalimentation, Parenteral OR Nutrition, Total Parenteral OR Intravenous Hyperalimentation OR Hyperalimentation, Intravenous OR Nutrition OR Nutrition Therapy)

3# TS=(tbi (traumatic brain injuries) OR tbis (traumatic brain injuries) OR traumatic encephalopathy OR injury, brain, traumatic OR traumatic encephalopathies OR encephalopathies, traumatic OR encephalopathy, traumatic OR tbi (traumatic brain injury) OR trauma*, brain OR brain trauma* OR traumatic brain injur* OR brain injur*, traumatic OR traumatic brain injury OR brain injur* OR Injur*, Brain OR Brain Laceration* OR Laceration*, Brain OR Cerebrovascular trauma OR Trauma, Cerebrovascular OR Trauma, Cerebrovascular OR Brain Vascular Trauma OR head injury OR Craniocerebral Trauma OR Cerebrovascular Trauma OR diffuse axonal injur*)

4# TS=((clinical AND trial) OR clinical trials as topic OR clinical trial OR random* OR random allocation OR therapeutic use)

5# ((#1 OR #2) AND #3) AND #4

6# TS=(child OR children OR pediatric* OR infant* OR infant*, newborn OR newborn infant* neonat*)

7# TS=(animal* OR mice OR rat OR rabbit* )

8# ((#5) NOT #6) NOT #7
