# Supplementary figures and images for: The Efficacy of Parenteral Nutrition and Enteral Nutrition Supports in Traumatic Brain Injury: A Systemic Review and Network Meta-Analysis
Source: Emerg Med Int. 2023 Apr 20;2023:8867614. doi: 10.1155/2023/8867614 (PMC10139805; doi:10.1155/2023/8867614)

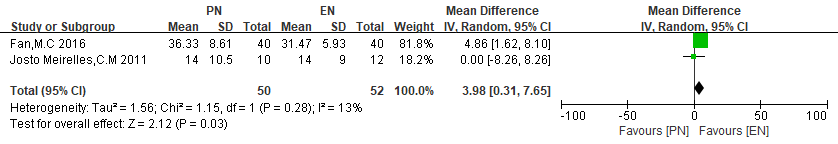

Supplement: Supplementary Materials — Figure S1: LOS in the ICU between the EN and PN groups. Figure S2: Incidence of stress ulcer between the EN and PN groups. Figure S3: Nitrogen balance during 0-1 d (A), 3 d (B), 7 d (C), and 10-11 d (D) between the EN and PN groups. Figure S4: Nitrogen balance during 7 d (A) and 11–14 d (B) between the EN + PN and EN groups. Document S1: Details of the search strategy. [file 8867614.f1.zip › Figure S1 (1).png]

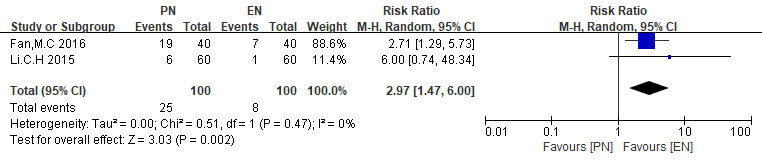

Supplement: Supplementary Materials — Figure S1: LOS in the ICU between the EN and PN groups. Figure S2: Incidence of stress ulcer between the EN and PN groups. Figure S3: Nitrogen balance during 0-1 d (A), 3 d (B), 7 d (C), and 10-11 d (D) between the EN and PN groups. Figure S4: Nitrogen balance during 7 d (A) and 11–14 d (B) between the EN + PN and EN groups. Document S1: Details of the search strategy. [file 8867614.f1.zip › Figure S2 (1).png]

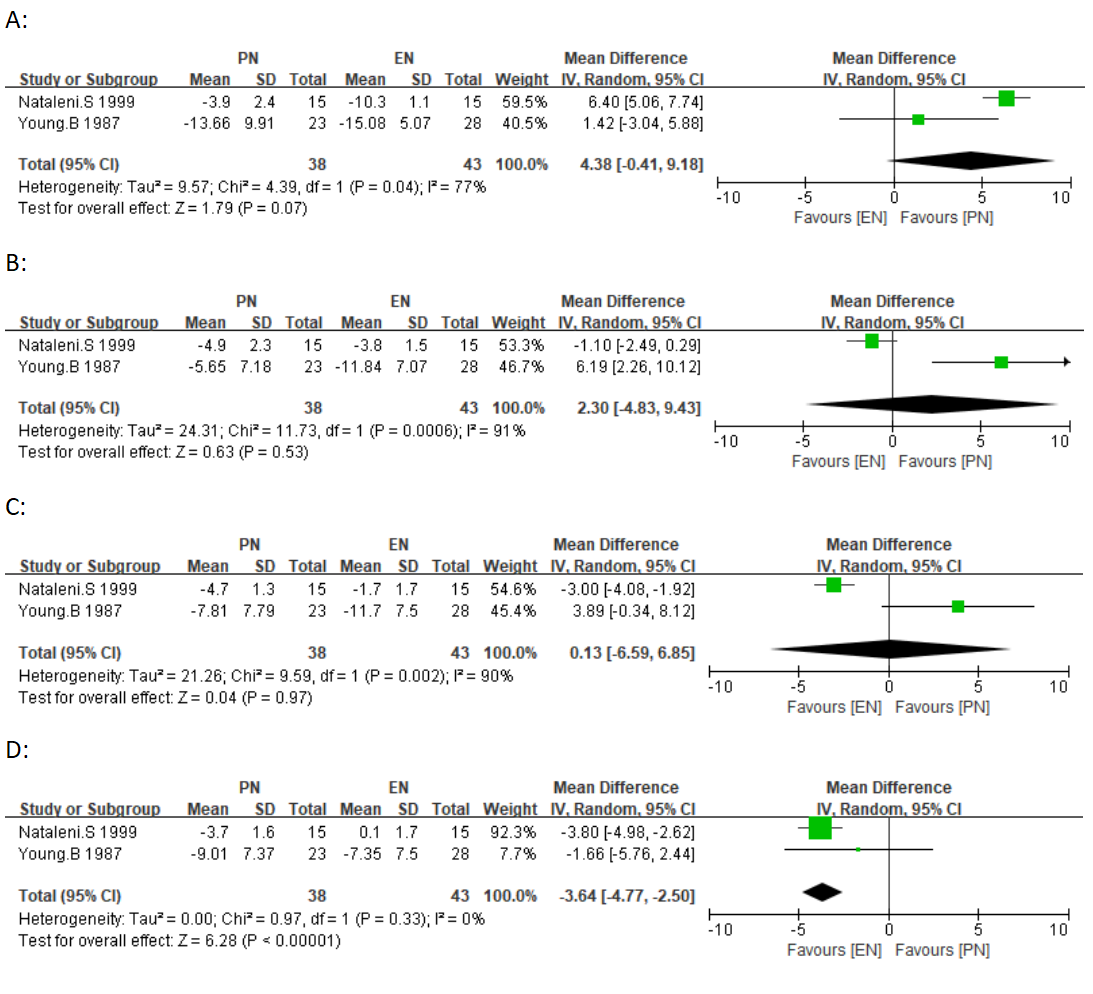

Supplement: Supplementary Materials — Figure S1: LOS in the ICU between the EN and PN groups. Figure S2: Incidence of stress ulcer between the EN and PN groups. Figure S3: Nitrogen balance during 0-1 d (A), 3 d (B), 7 d (C), and 10-11 d (D) between the EN and PN groups. Figure S4: Nitrogen balance during 7 d (A) and 11–14 d (B) between the EN + PN and EN groups. Document S1: Details of the search strategy. [file 8867614.f1.zip › Figure S3 (1).png]

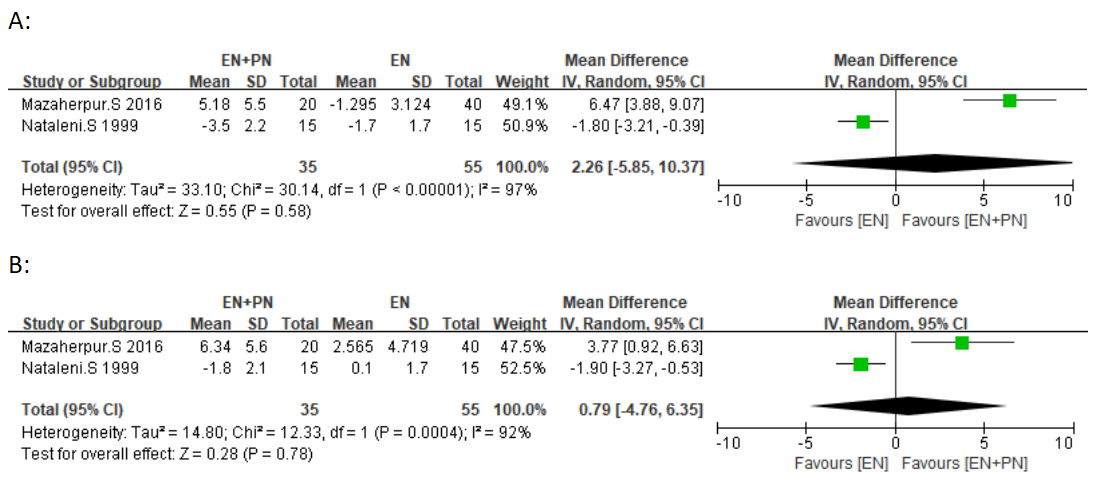

Supplement: Supplementary Materials — Figure S1: LOS in the ICU between the EN and PN groups. Figure S2: Incidence of stress ulcer between the EN and PN groups. Figure S3: Nitrogen balance during 0-1 d (A), 3 d (B), 7 d (C), and 10-11 d (D) between the EN and PN groups. Figure S4: Nitrogen balance during 7 d (A) and 11–14 d (B) between the EN + PN and EN groups. Document S1: Details of the search strategy. [file 8867614.f1.zip › Figure S4 (1).png]
